# The influence of anaesthetic choice on seizure duration of electroconvulsive therapy; etomidate versus methohexital

**DOI:** 10.1186/s12871-022-01745-y

**Published:** 2022-07-05

**Authors:** Laila Chomrikh, Mustafa Ahmadi, T. Martijn Kuijper, Joris J. B. van der Vlugt, Seppe J. H. A. Koopman

**Affiliations:** 1 Department of Anaesthesiology, Haaglanden Medical Centre, the Hague, the Netherlands; 2 Department of Anaesthesiology, Erasmus University Medical Centre, Rotterdam, the Netherlands; 3 Maasstad Academy, Maasstad Hospital, Rotterdam, the Netherlands; 4 Department of Psychiatry, Antes Parnassiagroep, Rotterdam, the Netherlands; 5 Department of Anaesthesiology, Maasstad Hospital, Maasstadweg 21, 3079 DZ Rotterdam, the Netherlands

**Keywords:** Electroconvulsive therapy, Affective disorder, Etomidate, Methohexital, Seizure duration

## Abstract

**Background:**

Many of the anaesthetic drugs used for electroconvulsive therapy have anticonvulsant properties and may influence efficacy of electroconvulsive therapy. With this study we aim to provide more information on the effect of etomidate and methohexital on seizure duration. We explore the relationship between induction drug, motor and electroencephalography seizure duration. Moreover, we study the relationship of seizure duration and number of therapies.

**Methods:**

In this retrospective study we collected data from patient records from 2005 until 2016. Inclusion criteria were the use of etomidate and/or methohexital and documentation of dosage, electroconvulsive therapy dosage and seizure duration. Exclusion criteria were missing data on either induction drug, dosage or seizure duration.

**Results:**

Thirty seven patients were analysed. The mean age was 52 years and seventy six percent were female. Most patients were suffering from affective disorders (81%). Motor and electroencephalography seizure duration were analysed in 679 and 551 electroconvulsive therapies, respectively. Compared to methohexital, motor and electroencephalography seizures under etomidate were 7 and 13 s longer, respectively. Furthermore, there was a negative association between seizure duration and number of treatment and a negative association between seizure duration and electroconvulsive therapy dosage.

**Conclusions:**

This study demonstrates significant longer motor and electroencephalography seizure duration using etomidate compared to methohexital. Etomidate might therefore increase the effectiveness of electroconvulsive therapy. Moreover, we observed a negative association between seizure duration, number of treatment and electroconvulsive therapy dosage. With this study we contribute to the available literature comparing methohexital and etomidate as induction agents for electroconvulsive therapy.

**Supplementary Information:**

The online version contains supplementary material available at 10.1186/s12871-022-01745-y.

## Introduction

Electroconvulsive therapy (ECT) is a safe and effective treatment of severe and medication resistant psychiatric disorders such as depression and mania [1]. An electrical current is applied to the brain via transcutaneous electrodes while the patient is under general anaesthesia. This electrical current results in a generalized (motor) seizure accompanied by an acute cardiovascular response due to activation of the autonomic nervous system.

Despite uncertainties in the literature regarding good indicators for the efficacy of ECT, there is evidence that there is a relationship between the seizure duration and the effectiveness of ECT [2–5]. Many of the anaesthetic drugs used for ECT have anticonvulsant properties and may alter seizure parameters, e.g. the decrease of the duration of the induced seizure activity. They might therefore decrease the effectiveness of ECT.

Methohexital, a short-acting barbiturate, remains the most widely used anaesthetic for ECT due to its potential for long seizure duration and is recommended as drug of first choice for ECT by the American Psychiatry Association [6–8]. Despite this fact, etomidate gained popularity as an anaesthetic agent for ECT, due to its positive effects on electroencephalography (EEG) and motor seizure duration. Furthermore, it has a fast onset of action, short duration of effect and offers haemodynamic stability [8, 9]. The use of etomidate is associated with longer EEG and motor seizure durations. This might be because the absence of anticonvulsant effect of etomidate. Thus, it could be the induction drug of choice in patients experiencing inadequate seizure activity [6, 7, 10, 11]. Furthermore, it is described as the only induction agent that may reduce ECT dosage [6].

There are a limited number of studies comparing methohexital and etomidate in the use for ECT and these data are inconsistent [10, 12–15]. Some studies show no difference between etomidate and methohexital. Others show longer seizure duration and reduction of ECT dosage in favour of etomidate. Nonetheless, superiority or inferiority over methohexital could not be demonstrated with the presently available literature [8, 10, 12–14].

### Study aim

In our hospital either methohexital or etomidate is used in patients undergoing an ECT. The choice of induction drug depends on the preference of the anaesthesiologist. In the same patient, etomidate and methohexital might both have been used. This minimizes potential confounders and facilitates a ‘cross-over’ design.

With the present study we aim to provide more information on the effectiveness of etomidate compared to methohexital on ECT parameters. We will explore the relationship between induction drug and seizure duration measured by both motor seizure activity and EEG. Moreover, we will study the relationship of seizure duration and number of treatments.

## Material and methods

### Study design

The present study is a retrospective analysis of electroconvulsive therapy performed in our clinical institution from 2005 until 2016. The study was approved by the medical ethical committee (Toetsingscommissie Wetenschappelijk Onderzoek Rotterdam en omstreken; TWOR, 2016–61) and the need for written informed consent was waived. This study was performed in accordance with the Declaration of Helsinki. Hemodynamic data, induction drug and dosage, duration of seizure (both motor and EEG seizures) and demographic data were all recorded in patient records.

We included patients receiving electroconvulsive therapy between 2005 and 2016. Patients were included if they received etomidate and/or methohexital and if dosage, ECT dosage and duration of seizure were recorded. Patients with missing data on either induction drug, dosage or seizure duration were excluded.

In our hospital either methohexital or etomidate is used in patients undergoing an ECT. All anaesthesiologists working in our hospital are regularly assigned to cover the ECT service. The choice of induction drug depends on the preference of the anaesthesiologist and is decided independently of seizure and characteristics or psychiatrist preferences.

### Outcome

We explored the relationship between induction drug and seizure duration measured by both motor and EEG seizure activity. Motor seizure duration was defined as time from electrical stimulation to resolution of clonic activity in an isolated limb. EEG seizure duration was the time from delivery of electrical stimulus to postictal EEG suppression.

Furthermore, we studied the relationship between seizure duration and induction drug dosage and number of past treatments.

### Data collection

Data was collected from the hospital records (both paper and electronic). Baseline data included age, sex, number of ECT sessions, psychiatric diagnoses and diagnosis of kidney failure, cerebrovascular accident or epilepsy. In addition, we collected the subsequent data per ECT session; anesthetic agent and dosage used per therapy, opioid use, antihypertensive drug use, benzodiazepine use, antipsychotic drug use, tricyclic antidepressant use, uni- or bilateral stimulation, impedance, pulse width of the electrical stimulus, frequency used, ECT dosage, motor and EEG seizure duration and hemodynamics (systolic and diastolic pressure, heart rate). Because of differences in schedule time of the ECT between different days, we also documented the day at which the ECT took place. We performed a stratified analysis of these data.

The data was handled in accordance with the Dutch Data Protection Act and the privacy regulation of the Maasstad Hospital. Each patient was allocated an unique identification number. Data was directly entered into a secured online database management system. Permission for this study was granted by the Institutional Review Board.

### Statistical analysis

#### Descriptive statistics

Descriptive statistics were presented as counts and percentages (dichotomous variables) or means and standard deviations ((sd); normally distributed continuous variables). Non-normally distributed, continuous variables were described as medians with interquartile range.

### Relationship between induction drug and seizure duration

The relationship between seizure duration and induction agent was modeled by repeated measures analysis using a linear mixed model with random intercept for patient and random slopes for treatment number and induction drug. Measurements up to 365 days after start of convulsion therapy were included. To allow for easier interpretation of parameters, covariate ECT dosage was centered at 100%, while covariates drug dosage for etomidate and methohexital were centered at their most frequently prescribed dosages of 20 mg and 100 mg respectively. Covariates include a dummy variable for induction drug (etomidate, reference methohexital), number of treatment, ECT dosage (range 0–200%, centred at 100%) and induction drug dosage, centred at 20 mg (etomidate) and 100 mg (methohexital). For both outcomes (motor seizure activity and seizure activity on EEG), the following model building strategy was undertaken. First, relationships between outcome and covariates number of treatment, ECT dosage, induction drug and dosage were graphically assessed by scatter plots. Secondly, saturated models were fit for each drug separately, including all interactions for number of treatment, dose and induction threshold. Starting with the three-way interaction, interaction terms were sequentially removed in case the likelihood ratio-test Chi2-statistic was not significant for that term (which proved to be the case for all the interaction terms). Thirdly, models for both drugs were combined, starting with all two-way interactions between induction drug and covariates number of treatment, ECT dosage and dosage (the drug-dosage interaction term being forced into the model for interpretation). Again, interactions terms were sequentially removed based on the likelihood ratio-test Chi2-statistic. All models were fit with a random intercept for patient and random slopes for treatment occasion and an unstructured covariance for the random effects under full maximum likelihood. For the final models, the addition of a random slope for induction drug and allowing for heterogeneity in residual errors by drug were tested by the likelihood test under restricted maximum likelihood. These resulted in a small but significant improvement in model fit and were hence incorporated in the final models. Various sensitivity analyses were performed to ensure validity of our results.

*P*-values < 0.05 were considered statistically significant.

## Results

### Demographics

**Fig. 1:**
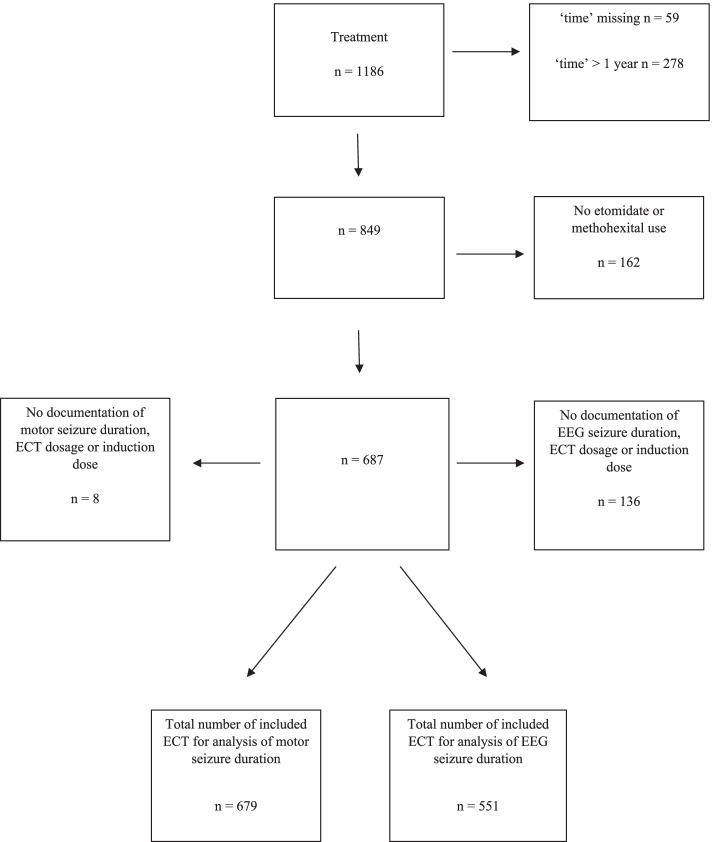
Flowchart. Measurements up to 365 days after start of electroconvulsive therapy were included. We excluded other anaesthetic induction agents than etomidate or methohexital. We excluded patients with missing data of motor or EEG seizure duration, ECT dosage and induction dose as well. A total number of 679 and 551 electroconvulsive therapies were included for analysis regarding motor seizure duration and EEG seizure duration respectively. ECT electroconvulsive therapy; EEG electroencephalography; n number

From 2005 until 2016, 102 patients were treated with ECT. Yet, we only found complete hospital documentation of 37 patients (see Fig. 1).

A total number of 37 patients were analysed. The mean age of the patients was 52.3 years (median 50, sd 13.7, interquartile range (IQR) 44–63 (number (*n*) = 37). Seventy six percent were female. Most patients were suffering from affective disorders (81%) and three patients (8%) were diagnosed with schizophrenia. Psychiatric diagnosis was unclear in four patients (11%). See Table 1 for the demographic data. Medication use and comorbidity are described in Table 1. Benzodiazepines and antipsychotic drugs were the most frequently used drugs. We also documented the number of patients with missing information on their medication use.

**Table 1:** Demographic characteristics

Characteristic	All patients (*n* = 37)	Methohexital only (*n* = 13)	Etomidate only (*n* = 8)	Methohexital and etomidate (*n* = 16)
Age (year), mean (sd)	52.3 (13.7)	53.5 (16.6)	47.4 (16.5)	53.9 (9.3)
Gender, n (%)				
Male	9 (24)	3 (23)	0 (0)	6 (38)
Female	28 (76)	10 (77)	8 (100)	10 (63)
Weight (kg), mean (sd)	77.8 (16.2)	71.7 (9.9)	86.2 (21.9)	78.5 (16.1)
Diagnosis, n (%)				
Affective disorder	30 (81)	12 (92)	7 (88)	11 (69)
Schizophrenia	3 (8)	1 (8)	0 (0)	2 (12)
Unclear	4 (11)	0 (0)	1(12)	3 (19)
Comorbidity, n (%)				
CVA/TIA	1 (3)	1 (8)	0 (0)	0 (0)
Epilepsy	2 (6)	0 (0)	1 (13)	1 (7)
Renal impairment (GFR < 60)	4 (11)	1 (8)	2 (25)	1 (7)
Medication use, n (%)				
Antihypertensive drugs	11 (31)	4 (31)	3 (38)	4 (27)
Antipsychotic drugs	25 (71)	9 (69)	5 (63)	11 (79)
Benzodiazepine	28 (80)	11 (85)	7 (88)	10 (71)
Tricyclic antidepressant	11 (31)	4 (31)	2 (25)	5 (36)
Opioids	0 (0)	0 (0)	0 (0)	0 (0)

*yr* year, *sd* standard deviation, *n* number, *ECT* electroconvulsive therapy, *EEG* electroencephalography, *CVA* cerebrovascular accident, *TIA* transient ischemic attack, *GFR* glomerular filtration rate, *Std. error* standard error, *CI* 95% confidence interval

The mean induction drug dosage use for methohexital and etomidate was 100.7 (sd between and within patients: 17.6 and 21.9) mg and 22.4 (sd between and within patients: 4.2 and 5.7) mg respectively. Calculating the mean dosage per weight amounted to 1.4 mg/kg (methohexital) and 0.3 mg/kg (etomidate).

### ECT parameters

A total number of 679 and 551 ECT were included for analysis regarding motor seizure duration and EEG seizure duration respectively.

Patients received an average of 23 therapies in the first year following the first ECT. Methohexital was used in 49.5% of these treatments. Etomidate was used in 31.5% of the treatments. Propofol was used in three treatments (0.3%). In 18.7% of the treatments, induction drug was not reported. These patients were not included in the analysis.

During the first year of treatment, *n* = 13 patients received only methohexital, *n* = 8 patients only etomidate and 16 patients received both induction agents. Descriptive data for these subgroups are presented in Table 1.

### Induction drug and seizure duration

Fixed effects coefficients for the final linear mixed model for seizure duration are shown in Table 2. A negative association of -0.27 (95% CI -0.51—-0.02) seconds for each subsequent treatment is observed between seizure duration and number of treatments. A negative association is observed between seizure duration and ECT dosage as well; -0.05 (95% CI -0.08—-0.01) seconds per percent increase in ECT dosage. Both induction agents show a negative dose–response relationship. For etomidate it is -0.36 (95% CI -0.64—-0.07) and for methohexital: -0.13 (95% CI -0.18—-0.09) seconds per milligram increase in dosage of induction drug. Overall, for the reference dosages of 20 mg and 100 mg for etomidate and methohexital respectively, seizures induced under etomidate had on average a longer duration compared to methohexital (6.69 (95% CI 1.43 – 11.95) seconds). Similar results were obtained for seizure duration on EEG. Seizures induced by etomidate were on average 13.40 (95% CI 8.38 – 18.42) seconds longer compared to methohexital at their respective reference dosages (Table 3).

**Table 2:** Relationship between motor seizure duration and induction drug

Motor seizure duration	Regression coefficient	Std. error	*p*	95% CI
Treatment number^a^	-0.27	0.13	0.04	-0.15—-0.02
ECT dosage^b^	-0.05	0.02	0.01	-0.08—-0.01
Etomidate^c^	6.69	2.68	0.01	1.43—11.95
Etomidate dose^d^	-0.36	0.15	< 0.001	-0.64—-0.07
Methohexital dose^d^	-0.13	0.02	< 0.001	-0.18—-0.09

^a^Seconds per each subsequent treatment

^b^Seconds per percent increase in ECT dosage

^c^Reference dose: etomidate 20 mg, methohexital 100 mg

^d^(seconds / mg increase in dosage)

*Std. error* standard error, 95% *CI* 95% confidence interval

**Table 3:** Relationship between EEG seizure duration and induction drug

EEG seizure duration	Regression coefficient	Std. error	*p*	95% CI
Treatment number^a^	-0.27	0.15	0.08	-0.57—0.03
ECT dosage^b^	-0.09	0.03	< 0.001	-0.15—-0.04
Etomidate^c^	13.40	2.56	< 0.001	8.38—18.42
Etomidate dose^d^	-0.58	0.21	0.01	-1.00—-0.16
Methohexital dose^d^	-0.12	0.03	< 0.001	-0.18—-0.05

^a^Seconds per each subsequent treatment

^b^Seconds per percent increase in ECT dosage

^c^Reference dose: etomidate 20 mg, methohexital 100 mg

^d^(seconds / mg increase in dosage)

*Std. error* standard error, 95% *CI* 95% confidence interval

As a sensitivity analysis, we also fitted the regression models on the subset of *n* = 16 patients that received both induction agents during the first year of treatment and found similar results for the effect of etomidate on seizure duration (supplemental tables 1a and 1b). Adjustment for induction agent by taking the dosage in milligrams per kilograms rather than using the raw dosage in milligrams also led to similar results, both on the complete dataset (supplemental tables 2a and 2b) and on the subset of *n* = 16 patients that received both induction agents (supplemental tables 3a and 3b). In addition, we fitted models adjusting for additional potential confounders age (centered at the group mean), gender, electrode placement (unilateral vs bilateral) and concomitant use of antipsychotic medication, tricyclic antidepressants and benzodiazepines (included as time-varying covariates) that can be found in supplemental tables 4a and 4b. Analyses taking only into consideration only those pairs of subsequent measurements at which a switch between induction agent occurred have been included in supplemental tables 5a and 5b. All these sensitivity analyses led to the same results.

## Discussion

In our study, we have shown that etomidate is associated with a long seizure duration (both EEG and motor) compared to methohexital. Furthermore, there is a negative dose–response relationship with the use of both induction agents. We also demonstrated a negative association between seizure duration and number of treatment and between seizure duration and ECT dosage.

Literature regarding the optimal induction drug for ECT is inconsistent. Our findings differ from a study by Eser et al. [12]. They performed a retrospective analysis of 5482 ECTs in 455 patients treated between 1995 and 2003. There was no difference between induction with etomidate or methohexital in seizure duration [12, 13]. The mean dosage of administered methohexital and etomidate was higher compared to our patient population (130 mg and 32 mg compared to 100 mg and 20 mg, respectively) [16]. And to achieve adequate seizures, patients with higher ECT dosage received methohexital more frequently than other induction drugs. In our patient group, the choice for using methohexital or etomidate was not based on ECT dosage but rather on the preference of the attending anaesthesiologist. Switches in induction drug in one patient were thus at random. The higher drug dosage and the use of methohexital in patients with higher ECT dosage could be an explanation for the different results.

A more recent systemic review and meta-analysis of Singh and colleagues [8] included seventeen trials involving the use of etomidate, methohexital, thiopental and propofol as induction agents for ECT. Four trials compared etomidate with methohexital [13–15, 17].

A total of 84 ECT settings were included in this meta-analysis. The etomidate group showed a longer EEG and motor seizure duration of etomidate compared to methohexital (2.23 and 1.45 s, respectively). This difference was, however, not statistically significant. Thus, the study was underpowered to demonstrate superiority of etomidate over methohexital. Another study in line with our finding is the research of Avramov’s et al. [10, 14, 15]. They studied a small group (*n* = 10) of patients with chronic depression with a total of 90 ECTs in a randomized cross-over study comparing etomidate and methohexital. Their results showed longer durations of EEG and motor seizures after etomidate with no clear dose–effect relationship. The inverse relationship between seizure duration and number of treatment and ECT dosage is in line with a recent study by Luccarelli et al. [18].

Limitations of this study include the retrospective design and the limited number of patients (*n* = 37). Though there were 102 patients treated from 2005 until 2016, we only found hospital documentation of 37 patients. The large number of missing patient records might affect the internal validity of our study. Comparing baseline characteristics of patients excluded because of missing data, no differences were found. This decreases the risk of non-randomly distributed missing data. We therefore think that our result are valid, both internally as externally. Despite the large amount of missing data, we were able to show a significant result, thus our study was not underpowered. Another limitation is the retrospective design and the large study period. During this study period, treatment remained largely the same. Any treatment effect found in this study is less likely due to chances in treatment but more likely due to a true difference in seizure duration between etomidate and methohexital. Regarding our outcome measures, we were unable to study effectiveness of ECT. Unfortunately, in our database, no data on clinical outcome measures was available. We therefore used seizure duration as a proxy. Although there are uncertainties in the literature regarding good indicators for the efficacy of ECT, seizure duration is universally accepted as marker for effectiveness of ECT [2–5].

Another limitation is the mix between acute ECT treatments (twice a week) and maintenance ECT (1 every 2–5 weeks). The time between treatments can be expected to be of influence on the progression of seizure duration as well. It indeed differed between patients and also within patients over time in our dataset. In order to capture this relationship, a complex interaction between treatment number and time would be needed. During the model building phase we have explored whether including both treatment number, time and its interaction led to a better model fit than including treatment number or time alone. This was not the case, led to violations of the linearity assumption for treatment number and time and made the model harder to interpret. On the contrary, the model with only treatment number appeared to capture the data and trend quite well. We therefore decided to go with the simpler model. Furthermore, we carried out analyses in patients switching induction drug in subsequent treatment In both analyses estimates for the effect of induction agent were in line with the effects observed in the main analyses.

The dose of induction agents were chosen to minimize the effect of an induction agent on seizure duration while avoiding awareness. During each session the aim was to keep the induction dose as low as clinically possible. In practice, the induction dose was usually equal to previous sessions with the same induction drug. Data on post-ictal agitation and awareness in our centre are lacking. Expert opinion by psychiatrists in our hospital suggest no difference in agitation or awareness between different induction drugs. Unfortunately, we have no data on this. Furthermore, given the small sample size, our study is underpowered to detect a difference. Antiepileptic medication was not corrected for in the final model. Although mentioned in the guidelines as medication which affects the seizure duration, there is no reason to presume medication used differed between the two groups since patients were their own controls [11]. Flumazenil is not commonly used in our hospital but only on indication. This indication is irrespective of the induction drug and vice versa. There is no reason to presume a difference in flumazenil use between the two induction drugs.

A further limitation is the preference of anaesthetic choice by the attending anaesthesiologist. In our patient group, the choice for using methohexital or etomidate was not based on patient characteristics, ECT dosage or psychiatrist preference but rather on the preference of the attending anaesthesiologist. Given the variable results in literature and our national guideline regarding the best choice for induction drug in ECT patients, anaesthesiologists in our centre switched based on their own belief regarding the best choice. Switches in induction drug in one patient were thus at random. This might have affected the outcomes of the study. On the other hand, this means of choosing an induction agent can also be considered as a strength since etomidate and methohexital were both used in the same patients. This minimizes confounding by indication and facilitates a ‘cross-over’ design.

The strength of this study was the fact that we were able to include a good number of patients who were anesthetized with both etomidate and methohexital. This made the patients their own control, thus elimination several confounders. Furthermore, the choice of induction drug was based on the preference of the attending anaesthesiologist. This made confounding by indication less likely. We used this in our analysis by using linear mixed model and at the same time we could correct for confounders as ECT dosage, induction drug dosage and sensitization effect (number of treatments). We believe that, due to lack of confounding by indication, non-random loss-to-follow-up and an advanced statistical model, our results are valid. Although a difference of 6.7 s in motor seizure duration and 13.4 s in EEG seizure duration might not seem clinically relevant, it is a decrease of 23% and 39% of the average motor and EEG seizure duration respectively.

In conclusion, this study demonstrated a longer EEG and motor seizure duration with the use of etomidate in comparison to methohexital. Moreover, we observed a negative association between seizure duration and number of treatments and a negative association between seizure duration and ECT dosage. With this study we contribute to the available literature comparing methohexital and etomidate as induction agents for ECT. For a definite conclusion on the effect of induction drug on ECT treatment success, a controlled prospective study should be performed using clinical outcome measures.

## Supplementary Information

**Additional file 1: Supplemental Table 1a. **Relationship between motor seizure duration and induction drug. Adjustment for induction agent dose is performed by using the respective dosages in milligrams per kilogram. Both variables were centered at their means (taking into account the clustering of the data). **Supplemental Table 1b. **Relationship between EEG seizure duration and induction drug. Adjustment for induction agent dose is performed by using the respective dosages in milligrams per kilogram. Both variables were centered at their means (taking into account the clustering of the data). **Supplemental Table 2a. **Relationship between motor seizure duration and induction drug in *n*=16 patients who received both induction agents during the first year of treatment. **Supplemental Table 2b. **Relationship between EEG seizure duration and induction drug in *n*=16 patients who received both induction agents during the first year of treatment. **Supplemental Table 3a. **Relationship between motor seizure duration and induction drug in *n*=16 patients who received both induction agents during the first year of treatment. Adjustment for induction agent dose is performed by using the respective dosages in milligrams per kilogram. **Supplemental Table 3b. **Relationship between EEG seizure duration and induction drug in *n*=16 patients who received both induction agents during the first year of treatment. Adjustment for induction agent dose is performed by using the respective dosages in milligrams per kilogram. **Supplemental Table 4a. **Relationship between motor seizure duration and induction drug with adjustment for additional covariates age (centered at the group mean) and gender and time-varying covariates electrode placement (unilateral vs bilateral) and concomitant use of antipsychotic medication, tricyclic antidepressants and benzodiazepines. **Supplemental Table 4b. **Relationship between EEG seizure duration and induction drug with adjustment for additional covariates age (centered at the group mean) and gender and time-varying covariates electrode placement (unilateral vs bilateral) and concomitant use of antipsychotic medication, tricyclic antidepressants and benzodiazepines. **Supplemental Table 5a. **Sensitivity analysis for motor seizure duration analyzing only pairs of subsequent measurements at which a switch between induction agents occurred. A multilevel mixed model was fitted with a random intercept and slope for measurement pair and induction agent at the first level and a random intercept for patient at the second level. Only pairs for which the measurements were no more than 21 days apart were included. A number of 15 patients and 57 pairs of measurements were analyzed. **Supplemental Table 5b. **Sensitivity analysis for EEG seizure duration analyzing only pairs of subsequent measurements at which a switch between induction agents occurred. A multilevel mixed model was fitted with a random intercept and slope for measurement pair and induction agent at the first level and a random intercept for patient at the second level. Only pairs for which the measurements were no more than 21 days apart were included. A number of 15 patients and 55 pairs of measurements were analyzed.

## Data Availability

All data generated or analysed during this study are available upon reasonable request directed at the last author (Seppe SHA Koopman) and after permission of our legal department.
