# Supplementary material for: The influence of anaesthetic choice on seizure duration of electroconvulsive therapy; etomidate versus methohexital
Source: BMC Anesthesiol. 2022 Jul 5;22:206. doi: 10.1186/s12871-022-01745-y (PMC9254631; doi:10.1186/s12871-022-01745-y)
Supplement: Supplementary file 1 — Additional file 1: Supplemental Table 1a. Relationship between motor seizure duration and induction drug. Adjustment for induction agent dose is performed by using the respective dosages in milligrams per kilogram. Both variables were centered at their means (taking into account the clustering of the data). Supplemental Table 1b. Relationship between EEG seizure duration and induction drug. Adjustment for induction agent dose is performed by using the respective dosages in milligrams per kilogram. Both variables were centered at their means (taking into account the clustering of the data). Supplemental Table 2a. Relationship between motor seizure duration and induction drug in n=16 patients who received both induction agents during the first year of treatment. Supplemental Table 2b. Relationship between EEG seizure duration and induction drug in n=16 patients who received both induction agents during the first year of treatment. Supplemental Table 3a. Relationship between motor seizure duration and induction drug in n=16 patients who received both induction agents during the first year of treatment. Adjustment for induction agent dose is performed by using the respective dosages in milligrams per kilogram. Supplemental Table 3b. Relationship between EEG seizure duration and induction drug in n=16 patients who received both induction agents during the first year of treatment. Adjustment for induction agent dose is performed by using the respective dosages in milligrams per kilogram. Supplemental Table 4a. Relationship between motor seizure duration and induction drug with adjustment for additional covariates age (centered at the group mean) and gender and time-varying covariates electrode placement (unilateral vs bilateral) and concomitant use of antipsychotic medication, tricyclic antidepressants and benzodiazepines. Supplemental Table 4b. Relationship between EEG seizure duration and induction drug with adjustment for additional covariates age (centered at the grou [file 12871_2022_1745_MOESM1_ESM.docx]

**Supplemental files**

**Supplemental Table 1a.** Relationship between motor seizure duration and induction drug. Adjustment for induction agent dose is performed by using the respective dosages in milligrams per kilogram. Both variables were centered at their means (taking into account the clustering of the data).

| Motor seizure duration | Regression coefficient | Std. error | *P* | 95% CI |
| --- | --- | --- | --- | --- |
| Treatment number ^+^ | -0.28 | 0.13 | 0.03 | -0.53 - -0.03 |
| ECT dosage ^$^ | -0.05 | 0.02 | 0.01 | -0.08 - -0.01 |
| Etomidate ^#^ | 6.57 | 2.73 | 0.02 | 1.23 - 11.91 |
| Etomidate dose * | -27.70 | 12.40 | 0.03 | -51.99 - -3.40 |
| Methohexital dose * | -10.5 | 1.80 | <0.001 | -14.03 - -6.98 |

^+^Seconds per each subsequent treatment; ^$^ Seconds per percent increase in ECT dosage; ^#^Reference dose: etomidate 0.28 mg/kg, methohexital 1.36 mg/kg; *(seconds / mg increase in dosage)

Std. error: standard error; 95% CI: 95% confidence interval

**Supplemental Table 1b.** Relationship between EEG seizure duration and induction drug. Adjustment for induction agent dose is performed by using the respective dosages in milligrams per kilogram. Both variables were centered at their means (taking into account the clustering of the data).

| EEG seizure duration | Regression coefficient | Std. error | *p* | 95% CI |
| --- | --- | --- | --- | --- |
| Treatment number ^+^ | -0.28 | 0.15 | 0.06 | -0.57 - 0.01 |
| ECT dosage ^$^ | -0.10 | 0.03 | <0.001 | -0.15 - -0.05 |
| Etomidate ^#^ | 14.34 | 3.45 | <0.001 | 7.59 - 21.10 |
| Etomidate dose * | -30.89 | 18.82 | 0.10 | -67.80 - 6.01 |
| Methohexital dose * | -8.11 | 2.59 | <0.01 | -13.18 - -3.03 |

^+^Seconds per each subsequent treatment; ^$^ Seconds per percent increase in ECT dosage; ^#^Reference dose: etomidate 0.28 mg/kg, methohexital 1.36 mg/kg; *(seconds / mg increase in dosage)

Std. error: standard error; 95% CI: 95% confidence interval

**Supplemental Table 2a.** Relationship between motor seizure duration and induction drug in n=16 patients who received both induction agents during the first year of treatment.

| Motor seizure duration | Regression coefficient | Std. error | *p* | 95% CI |
| --- | --- | --- | --- | --- |
| Treatment number ^+^ | -0.02 | 0.17 | 0.89 | -0.36 - 0.32 |
| ECT dosage ^$^ | -0.06 | 0.04 | 0.10 | -0.14 - 0.01 |
| Etomidate ^#^ | 7.56 | 3.08 | 0.01 | 1.53 - 13.60 |
| Etomidate dose * | -0.36 | 0.16 | 0.03 | -0.67 - -0.04 |
| Methohexital dose * | -0.15 | 0.03 | <0.001 | -0.22 - -0.08 |

^+^Seconds per each subsequent treatment; ^$^ Seconds per percent increase in ECT dosage; ^#^Reference dose: etomidate 20 mg, methohexital 100 mg; *(seconds / mg increase in dosage)

Std. error: standard error; 95% CI: 95% confidence interval

**Supplemental Table 2b.** Relationship between EEG seizure duration and induction drug in n=16 patients who received both induction agents during the first year of treatment.

| EEG seizure duration | Regression coefficient | Std. error | *p* | 95% CI |
| --- | --- | --- | --- | --- |
| Treatment number ^+^ | 0.23 | 0.20 | 0.27 | -0.17 - 0.62 |
| ECT dosage ^$^ | -0.22 | 0.06 | <0.001 | -0.33 - -0.11 |
| Etomidate ^#^ | 12.82 | 2.86 | <0.001 | 7.23 - 18.42 |
| Etomidate dose * | -0.47 | 0.22 | 0.03 | -0.91 - -0.04 |
| Methohexital dose * | -0.18 | 0.05 | <0.001 | -0.28 - -0.09 |

^+^Seconds per each subsequent treatment; ^$^ Seconds per percent increase in ECT dosage; ^#^Reference dose: etomidate 20 mg, methohexital 100 mg; *(seconds / mg increase in dosage)

Std. error: standard error; 95% CI: 95% confidence interval

**Supplemental Table 3a.** Relationship between motor seizure duration and induction drug in n=16 patients who received both induction agents during the first year of treatment. Adjustment for induction agent dose is performed by using the respective dosages in milligrams per kilogram.

| Motor seizure duration | Regression coefficient | Std. error | *p* | 95% CI |
| --- | --- | --- | --- | --- |
| Treatment number ^+^ | 0.00 | 0.16 | 0.99 | -0.31 - -0.32 |
| ECT dosage ^$^ | -0.07 | 0.04 | 0.07 | -0.14 - -0.01 |
| Etomidate ^#^ | 8.14 | 3.16 | 0.01 | 1.96 - 14.33 |
| Etomidate dose * | -29.3 | 13.75 | 0.03 | -56.24 - -2.35 |
| Methohexital dose * | -13.34 | 2.99 | <0.001 | -19.20 - -7.49 |

^+^Seconds per each subsequent treatment; ^$^ Seconds per percent increase in ECT dosage; ^#^Reference dose: etomidate 0.28 mg/kg, methohexital 1.36 mg/kg; *(seconds / mg increase in dosage)

Std. error: standard error; 95% CI: 95% confidence interval

**Supplemental Table 3b.** Relationship between EEG seizure duration and induction drug in n=16 patients who received both induction agents during the first year of treatment. Adjustment for induction agent dose is performed by using the respective dosages in milligrams per kilogram.

| EEG seizure duration | Regression coefficient | Std. error | *p* | 95% CI |
| --- | --- | --- | --- | --- |
| Treatment number ^+^ | 0.20 | 0.19 | 0.31 | -0.18 - 0.58 |
| ECT dosage ^$^ | -0.23 | 0.06 | <0.001 | -0.34 - -0.13 |
| Etomidate ^#^ | 14.71 | 4.02 | <0.001 | 6.83 - 22.59 |
| Etomidate dose * | -25.49 | 19.12 | 0.18 | -62.96 - 11.98 |
| Methohexital dose * | -16.42 | 4.62 | <0.001 | -25.48 - -7.37 |

^+^Seconds per each subsequent treatment; ^$^ Seconds per percent increase in ECT dosage; ^#^Reference dose: etomidate 0.28 mg/kg, methohexital 1.36 mg/kg; *(seconds / mg increase in dosage)

Std. error: standard error; 95% CI: 95% confidence interval

**Supplemental Table 4a.** Relationship between motor seizure duration and induction drug with adjustment for additional covariates age (centered at the group mean) and gender and time-varying covariates electrode placement (unilateral vs bilateral) and concomitant use of antipsychotic medication, tricyclic antidepressants and benzodiazepines.

| Motor seizure duration | Regression coefficient | Std. error | *p* | 95% CI |
| --- | --- | --- | --- | --- |
| Treatment number ^+^ | -0.27 | 0.12 | 0.04 | -0.51 - -0.02 |
| ECT dosage ^$^ | -0.04 | 0.02 | 0.03 | -0.08 - -0.00 |
| Etomidate ^#^ | 6.01 | 2.58 | 0.02 | -0.95 - 11.06 |
| Etomidate dose * | -0.37 | 0.15 | 0.01 | -0.66 - -0.09 |
| Methohexital dose * | -0.14 | 0.02 | <0.001 | -0.19 - -0.10 |
| Age | -0.32 | 0.14 | 0.02 | -0.60 - -0.05 |
| Female gender | -6.88 | 4.21 | 0.10 | -15.13 - 1.36 |
| Bilateral placement | -5.83 | 2.64 | 0.03 | -11.01 - -0.65 |
| Antipsychotics |  |  |  |  |
| yes | -0.34 | 4.41 | 0.94 | -8.98 - 8.30 |
| unknown | 8.74 | 8.47 | 0.30 | -7.86 - 25.32 |
| TCA |  |  |  |  |
| yes | -2.46 | 4.83 | 0.61 | -11.93 - 7.02 |
| unknown | -12.72 | 13.06 | 0.33 | -38.33 - 12.89 |
| Benzodiazepines |  |  |  |  |
| yes | 4.30 | 4.33 | 0.32 | -4.18 - 12.88 |
| unknown | 2.79 | 9.99 | 0.78 | -16.78 - 22.36 |

^+^Seconds per each subsequent treatment; ^$^ Seconds per percent increase in ECT dosage; ^#^Reference dose: etomidate 20 mg, methohexital 100 mg; *(seconds / mg increase in dosage)

Std. error: standard error; TCA: Tricyclic antidepressants 95% CI: 95% confidence interval

**Supplemental Table 4b.** Relationship between EEG seizure duration and induction drug with adjustment for additional covariates age (centered at the group mean) and gender and time-varying covariates electrode placement (unilateral vs bilateral) and concomitant use of antipsychotic medication, tricyclic antidepressants and benzodiazepines.

| EEG seizure duration | Regression coefficient | Std. error | *p* | 95% CI |
| --- | --- | --- | --- | --- |
| Treatment number ^+^ | -0.26 | 0.16 | 0.09 | -0.57 - 0.05 |
| ECT dosage ^$^ | -0.09 | 0.03 | <0.01 | -0.14 - -0.38 |
| Etomidate ^#^ | 16.18 | 3.31 | <0.001 | 9.69 - 22.67 |
| Etomidate dose * | -0.44 | 0.22 | 0.04 | -0.87 - -0.13 |
| Methohexital dose * | -0.12 | 0.03 | <0.001 | -0.19 - -0.06 |
| Age | -0.32 | 0.18 | 0.08 | -0.68 - 0.04 |
| Female gender | -6.25 | 5.49 | 0.26 | -17.01 - 4.52 |
| Bilateral placement | -7.01 | 3.68 | 0.06 | -14.23 - 0.21 |
| Antipsychotics |  |  |  |  |
| yes | 5.18 | 5.68 | 0.36 | -5.96 - 16.32 |
| unknown | 12.49 | 14.21 | 0.38 | -15.38 - 40.36 |
| TCA |  |  |  |  |
| yes | -0.52 | 6.08 | 0.93 | -12.43 - 11.39 |
| unknown | -20.88 | 19.93 | 0.30 | -59.95 - 18.19 |
| Benzodiazepines |  |  |  |  |
| yes | 5.52 | 5.71 | 0.33 | -5.67 - 16.71 |
| unknown | 8.55 | 13.89 | 0.54 | -18.67 - 35.78 |

^+^Seconds per each subsequent treatment; ^$^ Seconds per percent increase in ECT dosage; ^#^Reference dose: etomidate 20 mg, methohexital 100 mg; *(seconds / mg increase in dosage)

Std. error: standard error; TCA: Tricyclic antidepressants; 95% CI: 95% confidence interval

**Supplemental Table 5a.** Sensitivity analysis for motor seizure duration analyzing only pairs of subsequent measurements at which a switch between induction agents occurred. A multilevel mixed model was fitted with a random intercept and slope for measurement pair and induction agent at the first level and a random intercept for patient at the second level. Only pairs for which the measurements were no more than 21 days apart were included. A number of 15 patients and 57 pairs of measurements were analyzed.

| Motor seizure duration | Regression coefficient | Std. error | *P* | 95% CI |
| --- | --- | --- | --- | --- |
| ECT dosage ^$^ | 0.00 | 0.06 | 0.93 | -0.11 - 0.12 |
| Etomidate ^#^ | 7.53 | 2.91 | 0.01 | 1.83 - 13.22 |
| Etomidate dose * | -0.48 | 0.40 | 0.23 | -1.27 - 0.30 |
| Methohexital dose * | -0.12 | 0.05 | 0.02 | -0.22 - -0.02 |

^+^Seconds per each subsequent treatment; ^$^ Seconds per percent increase in ECT dosage; ^#^Reference dose: etomidate 20 mg, methohexital 100 mg; *(seconds / mg increase in dosage)

Std. error: standard error; 95% CI: 95% confidence interval

**Supplemental Table 5b.** Sensitivity analysis for EEG seizure duration analyzing only pairs of subsequent measurements at which a switch between induction agents occurred. A multilevel mixed model was fitted with a random intercept and slope for measurement pair and induction agent at the first level and a random intercept for patient at the second level. Only pairs for which the measurements were no more than 21 days apart were included. A number of 15 patients and 55 pairs of measurements were analyzed.

| EEG seizure duration | Regression coefficient | Std. error | *p* | 95% CI |
| --- | --- | --- | --- | --- |
| ECT dosage ^$^ | -0.13 | 0.09 | 0.15 | -0.31 - 0.05 |
| Etomidate ^#^ | 7.87 | 3.76 | 0.04 | 0.51 - 15.24 |
| Etomidate dose * | 0.04 | 0.43 | 0.93 | -0.81 - 0.89 |
| Methohexital dose * | -0.22 | 0.09 | 0.01 | -0.40 - -0.05 |

^+^Seconds per each subsequent treatment; ^$^ Seconds per percent increase in ECT dosage; ^#^Reference dose: etomidate 20 mg, methohexital 100 mg; *(seconds / mg increase in dosage)

Std. error: standard error; 95% CI: 95% confidence interval
